# Extraosseous Osteosarcoma of the Esophagus: A Case Report

**DOI:** 10.1155/2010/907127

**Published:** 2010-03-04

**Authors:** Rodney E. Wegner, Kevin M. McGrath, James D. Luketich, David M. Friedland

**Affiliations:** ^1^UPMC Shadyside Hospital, Department of Radiation Oncology, University of Pittsburgh School of Medicine, 5230 Centre Avenue, Pittsburgh, PA 15232, USA; ^2^Division of Gastroenterology, Hepatology, and Nutrition, University of Pittsburgh School of Medicine, Pittsburgh, PA 15213, USA; ^3^Heart, Lung, and Esophageal Surgery Institute, University of Pittsburgh School of Medicine, Pittsburgh, PA 15213, USA; ^4^Department of Medical Oncology, University of Pittsburgh School of Medicine, Pittsburgh, PA 15213, USA

## Abstract

Extraosseous osteosarcoma (EOO) is a malignant mesenchymal neoplasm that is located in the soft tissues without direct attachment to the skeletal system and that produces osteoid, bone, or chondroid material. EOO is an extremely rare disease, accounting for only 1% of soft tissue sarcomas, and typically presents in either an extremity or the retroperitoneum. This paper presents the case of a 45-year-old Caucasian male with extraosseous osteosarcoma of the esophagus.

## 1. Case Report

**Figure 1:**
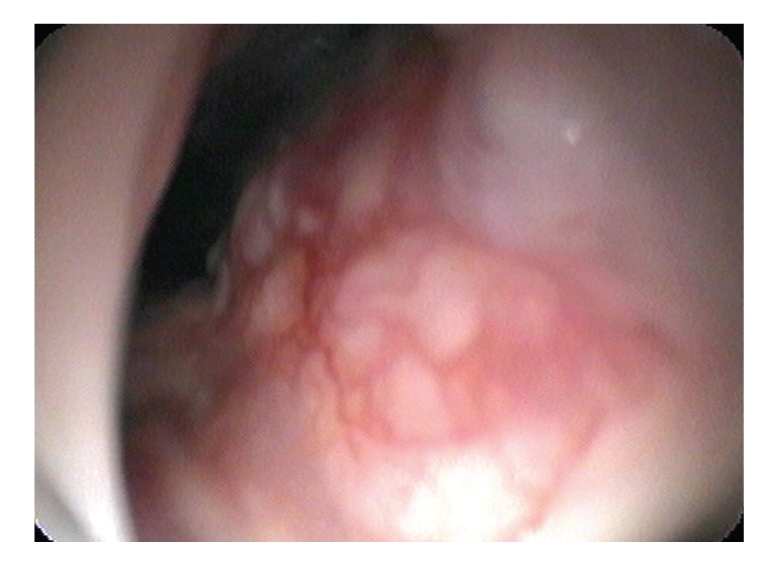
The proximal portion of the nodular tumor as seen on endoscopy of the esophagus. At time of this endoscopy, there was a severe stricture secondary to the tumor and a 6 mm scope was unable to be passed beyond it.

**Figure 2:**
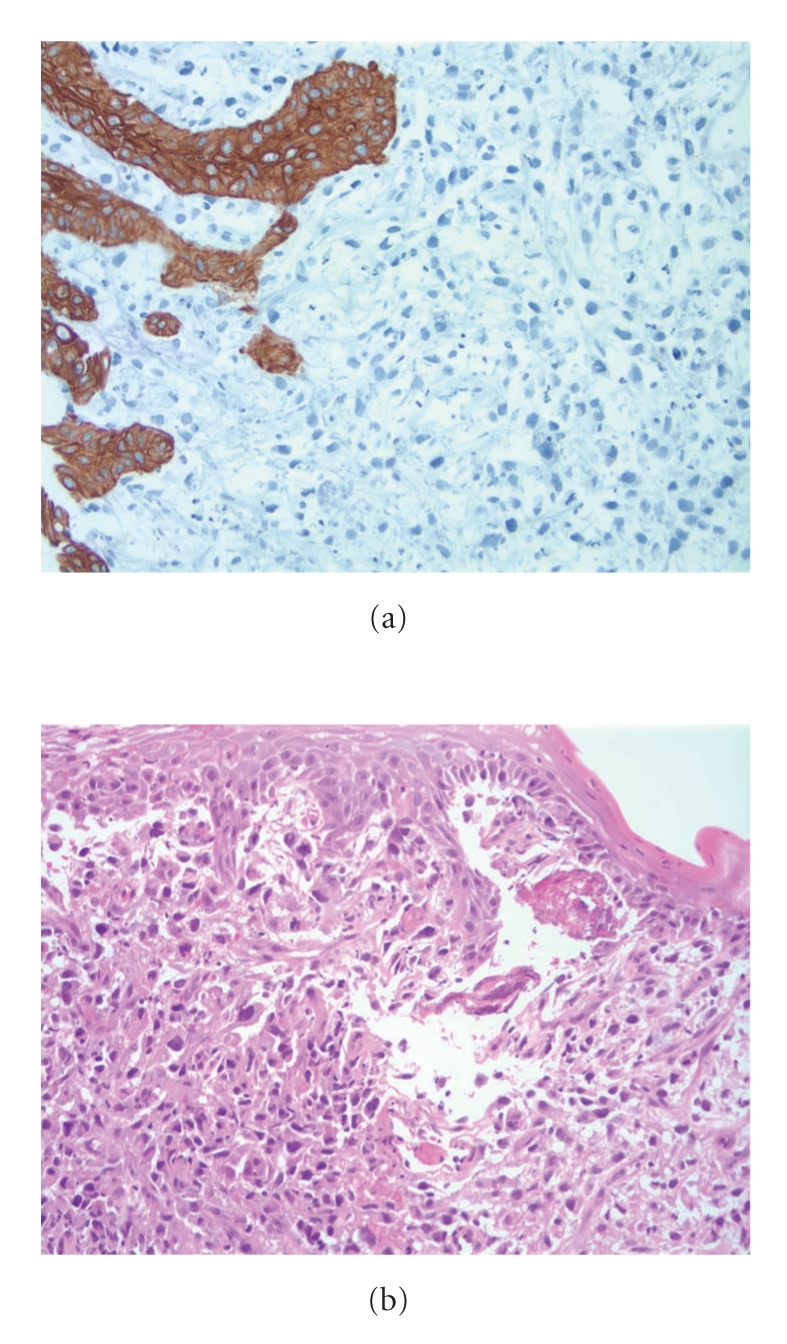
Pathology slide. (a) Tumor cells from the esophageal mass shown to be negative for pankeratin, with presence of actin being equivocal. (b) The submucosa of the esophagus contains mitotically active spindle cells and pleomorphic spindle cells with eosinophilic cytoplasm and focal areas containing eosinophilic stromal hyaline-like material, likely osteoid.

**Figure 3:**
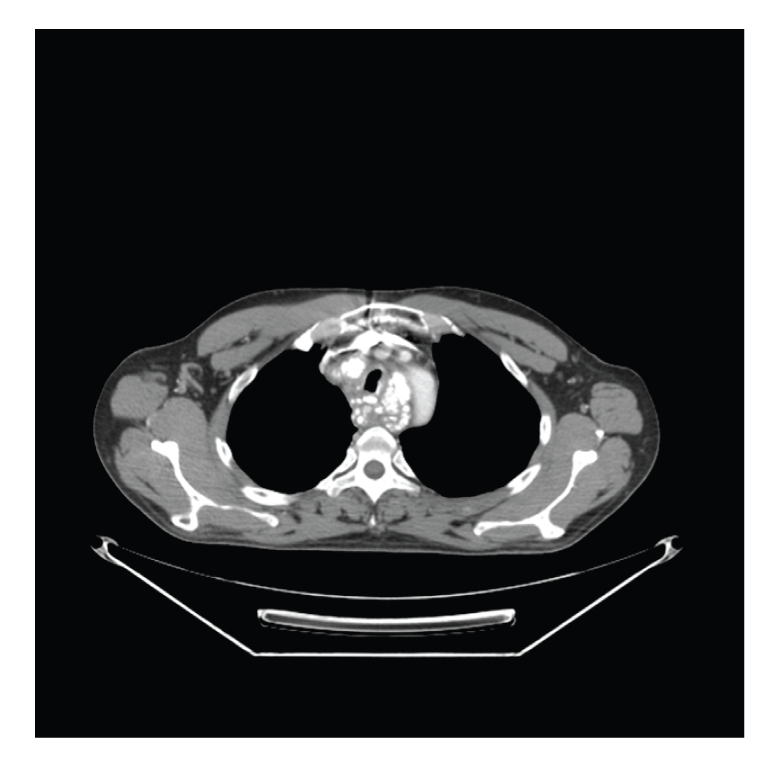
Lobulated, calcified esophageal mass at T4 level, as seen on CT scan. The mass almost completely surrounds the trachea and essentially obliterates the esophagus. Extensive calcification of a soft tissue mass and its presumed metastases should make one consider EOO as a possible diagnosis.

A 45-year-old Caucasian man first noticed a change in his normal state of health with some hoarseness in his voice. This hoarseness persisted for just over a week with no other associated symptoms. As the hoarseness resolved, the patient began to experience significant dysphagia to both hard and soft foods. Associated symptoms included nausea and regurgitation, but no emesis. He had also developed a new persistent cough, which was productive of clear mucus and sometimes scant hemoptysis. The patient reported fatigue, mild dyspnea on exertion, and a 10–15 pound weight loss over the ensuing 6–8 weeks. The patient was a lifetime nonsmoker and up until 3 years earlier had drank approximately 24 twelve ounce beers per week for several years. The family history was reviewed and was negative for any malignancy or familial syndromes with a predisposition for malignancy. The patient was seen by his primary care physician who ordered a chest CT scan, revealing multiple small polypoid lesions, all measuring less than 5 mm, along the wall of his distal trachea and proximal left main bronchus. There were also multiple enlarged calcified lymph nodes in the superior mediastinum. The work up continued with an esophagogastroduodenoscopy, which showed an ulcerated lesion extending from 20 to 30 cm from the incisors (Figure 1). Endoscopic ultrasound (EUS) was limited but did confirm a large mass in the right posterior margin of the upper esophagus. Biopsies were taken and showed mitotically active spindle cells and focal areas containing eosinophilic stromal hyaline-like material—osteoid. The tumor cells were noted to be vimentin positive, but negative for cytokeratin, desmin, CD117, Melanin, CD45, CD3 and CD20—further supporting the diagnosis of EOO (Figure 2). The patient underwent bronchoscopy showing multiple endobronchial masses; biopsies were taken and again showed pleomorphic spindle cells. The patient also had a palpable lymph node in his right supraclavicular region; this was biopsied, and pathology results were consistent with EOO. A PET/CT revealed an extensive, calcified, lobulated esophageal mass (Figure 3) with intense FDG avidity extending from T1 to T8, approximately 16 cm in length. In addition, he had bilateral FDG-avid level VI cervical lymph nodes, FDG-avid lymphadenopathy in the mediastinum, multiple bilateral pulmonary nodules, and calcified FDG-avid gastrohepatic lymph nodes. 

The patient underwent placement of an esophageal stent, which helped relieve his dysphagia. Medical oncology was consulted to determine the role of systemic therapy. The patient was seen, evaluated, and scheduled to receive 4 cycles of doxorubicin and cisplatin. Given the advanced disease present in the patient, chemotherapy consisting of doxorubicin and cisplatin was decided to be the best course of treatment. The patient went to the OR for placement of a port with repeat esophagoscopy. The tumor had increased considerably from the previous endoscopy and the patient was not able to be extubated due to significant local invasion of the tumor involving his entire trachea. He was eventually extubated and the patient and his family decided on placement in hospice care.

## 2. Discussion

EOO is a rare condition, comprising approximately 1% of all soft tissue sarcomas [1]. True EOO of the esophagus has only been reported in one patient back in the early 1980s [2]. In contrast to primary osteosarcoma, EOO tends to present at an older age (median age 50.7 years) [3]. More common anatomical sites for EOO include the lower extremities and retroperitoneum. Similar to osseous osteosarcoma, most patients with EOO will not have lymph node involvement. Interestingly, this case involved extensive lymph node involvement. Possible causes for EOO include radiation and trauma, neither being present in this case [4]. 

Most EOOs are high grade. Microscopically EOOs consist of neoplastic osteoid and bone, and frequently neoplastic cartilage. EOOs will stain positively for vimentin and there are now monoclonal antibodies to osteonectin and osteocalcin to help recognize skeletal and extraskeletal osteosarcomas. The above case involved areas of eosinophilic stromal hyaline-like material, interpreted by the pathologist as osteoid. The samples taken were also positive for osteonectin. The differential diagnosis of bone- and cartilage-forming soft tissue lesions can be long and sometimes difficult to narrow. Malignant lesions that can produce bone or cartilage include synovial sarcoma, epithelioid sarcoma, malignant fibrous histiosarcoma, liposarcoma, malignant melanoma, as well as others. Most of these neoplasms will have osteoid or bone in only a small portion of the tumor in a well-differentiated pattern. Oftentimes this is the only way to distinguish some of the above diagnoses from EOO [5]. 

Patients presenting with localized EOO have traditionally been treated with surgical resection and chemotherapy. Radiation has been used in the past with little success, and is now used sparingly for palliation [4]. Patients are usually treated with four cycles of doxorubicin-based chemotherapy. A study of outcomes in 60 patients with EOO showed only a 19% objective response rate to doxorubicin-based chemotherapy. Ifosfamide is sometimes used in combination with doxorubicin with reported response rates of 25%. Cisplatin has also been studied in the treatment of EOO with response rates of 13%. In patients treated with any of the above chemotherapies, approximately 40–50% will have progressive disease [6]. In the previously reported case of EOO of the esophagus, the patient was diagnosed with EOO after esophagogastrectomy with T-tube feeding jejunostomy. The patient died shortly after postoperative discharge from aspiration pneumonia. 

The overall prognosis for patients with EOO remains poor. Only about 1/3 of patients will survive for a long term. Many patients will have local recurrence and most will die of metastatic disease in 2-3 years [5]. Those patients presenting with metastatic disease, as in this case, have a median survival of 8 months [6].
